# Influence of the third molar presence on the thickness and height of the buccal cortical bone of the mandibular first and second molars

**DOI:** 10.4317/jced.60428

**Published:** 2023-06-01

**Authors:** Paulo-Márcio-de Mendonça Pinheiro, Karina-Maria-Salvatore Freitas, Renata-Cristina-Faria-Ribeiro de Castro

**Affiliations:** 1 DDS, MSc. São Leopoldo Mandic Dentistry Faculty, Campinas, Brazil; 2 DDS, MSc, PhD. Professor, Department of Orthodontics, Ingá University Center Uningá, Maringá, Brazil; 3 DDS, MSc, PhD. Professor, Renata Castro Institute, Inter and Multidisciplinary Dentistry, Campinas, Brazil

## Abstract

**Background:**

This study aimed to evaluate the influence of the presence of the third molars on the thickness and height of the buccal cortical bone of the first and second mandibular molars.

**Material and Methods:**

The retrospective cross-sectional observational sample consisted of 102 CBCTs of patients (mean age of 29 years), divided into two groups: G1: 51 patients (26 female; 25 male, mean age of 26 years) presenting the mandibular third molars and G2: 51 patients (26 female; 25 male, mean age of 32 years) with the absence of the mandibular third molars. The total and the cortical depth were evaluated at 4 and 6mm from the cementoenamel junction (CEJ). The total thickness of the buccal bone was evaluated in two horizontal reference lines located apically 6 mm and 11 mm from the CEJ. Statistical comparisons were performed with Mann Whitney and Wilcoxon tests.

**Results:**

In the comparison of buccal bone thickness and height between the groups, there was a statistical difference in tooth 36. In tooth 37 there was a statistical difference in the mesial root. For tooth 47, there was a statistical difference for the total thickness at 6mm, 11mm and 4mm. Concerning age, there was a tendency to decrease the values of these variables with increasing age.

**Conclusions:**

The mean values for buccal bone thickness, total and cortical depth of the mandibular molars were higher for patients with mandibular third molars because the buccal bone thickness of the mandibular molars increased in the posterior and apical direction.

** Key words:**Molar tooth, jaw, bone, orthodontic anchorage procedures, cone-beam computed tomography.

## Introduction

Over the last few years, with the greater use of extra-alveolar mini-implants, research has directed its measurements of buccal bone thickness to the roots of the mandibular molars and bone depth, since these mini-implants are thicker and longer than the inter-roots are installed parallel to the long axis of the molar roots (1-3). The bone region located buccally to the roots of the mandibular molars, also described as Mandibular Buccal Shelf (MBS), has been, in the last decade, the scope of several studies that described details of the bone anatomy to elucidate the best location for mini-implants installation. The place of installation of these devices is directly related to the biomechanics necessary for the correction of malocclusion (4). For extra-alveolar mini-implants (parallel to the long axis of the tooth), in addition to cortical thickness, the total thickness (horizontal direction), bone depth (vertical direction), and bone surface angulation were investigated (4,5), as well as the proximity of the mini-implant to the inferior alveolar nerve (1,2,6).

 Studies evaluating the thickness of the cortical bone in three dimensions and the depth of the bone in the buccal region of the mandibular molars are scarce, in addition, some used smaller samples: 32 adults (7); 12 children in mixed dentition (8); 30 adults (9); 30 adolescents (1), or evaluating specific ethnic groups (12 adults with Class III malocclusion, Asian descent) (4). The use of different methodologies was observed (measurements from the alveolar bone crest (2,4,7,10,112,4,7,10,11), or the cemetoenaml junction (CEJ) (1,5,8,9,12), even using molar root thirds (6,13) and, to date, have not evaluated whether the presence or absence of mandibular third molars would lead to differences in the thickness and height of the buccal cortex in the region of the mandibular first and second molars, a fact that motivated the carrying out this research.

This way, the objective of this study was to evaluate the influence of the presence of the third molars on the thickness and height of the buccal cortical bone of the first and second mandibular molars.

## Material and Methods

This research obtained approval from the Research Ethics Committee from the São Leopoldo Mandic Dentistry School, with number 2.813.597.

The retrospective cross-sectional observational sample consisted of 102 CBCTs (51 without mandibular third molars and 51 with mandibular third molars) of patients aged between 13.2 years and 59.2 years, with a mean age of 29.0 years (SD=12.3). The mean age of patients without mandibular third molars (M=32.0; SD=12.7) was higher than that of patients with mandibular third molars (M=26.0; SD=11.2) (*p*=0.013). The gender distribution was similar in both groups: 51% were female and 49% were male (Table 1).

**Table 1:**
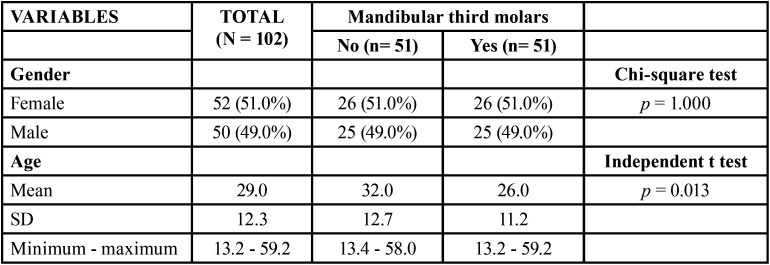
Sample profile and comparison of groups regarding gender and age.

The sample was divided into two groups: G1 with 51 tomographic images showing mandibular third molars and G2 with 51 tomographic images showing absence of mandibular third molars, randomly selected from the digital file of the Radiology Center of the São Leopoldo Mandic Dentistry School. CBCT scans were performed between June 2017 and January 2019. Random selection of patients was performed as follows: after selecting patients who met the selection criteria, these patients were numbered in order of age, separately by gender, so that, with the help of a random sequence generator (http://www.randomizer.org), a similar number of patients for each gender could be drawn.

1. Adult patients, without distinction of facial patterns and ethnicities, aged between 13.2 and 59.2 years;

2. CBCTs of the mandible or maxilla and mandible, from June 2017 to January 2019.

3. CBCTs with good image acquisition quality, same OP300 device (Instrumentarium Dental, Tuusula, Finland) found in the database of the Center of Radiology, São Leopoldo Mandic Dentistry School.

In the group of tomographic images with the absence of mandibular third molars, it is necessary to emphasize the limitation of the absence of an evaluation of the previous history, making it impossible to differentiate between absences due to agenesis or extractions.

After consulting the medical records of the patients selected in this study, it was possible to assess the exclusion criteria highlighted below:

1. Presence of periodontal disease

2. Presence of metallic restorations in mandibular premolars and molars

3. Dental absences, except the third molars

4. Presence of genetic syndromes

5. Facial trauma history

6. Previous orthognathic surgery

-Methods

CBCT scans were performed using an OP 300 CT scanner (Instrumentarium Dental, Tuusula, Finland) with the following image acquisition parameters: 90 kV, 10 mA and FoV covering the entire mandible. Each exam was converted into DICOM (digital imaging and communications in medicine) format and was processed by the program that came with the tomograph.

For the proper visualization of the sections of the buccal bone region of the mandibular molars for the quantitative and qualitative evaluation of this bone, some procedures were necessary for orientation in the three planes (axial, sagittal and coronal), during visualization through the software interface (9).

**Figure 1:**
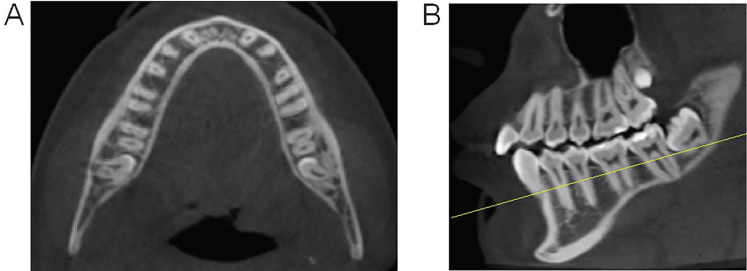
Cone-beam computed tomography images, in the axial and sagittal planes, illustrative of the orientation of the axial plane, for the posterior orientation of the sagittal and coronal planes. A) Axial plane passing through the furcation of the right mandibular first molar, the furcation of the left mandibular first molar, and the furcation of the right mandibular second molar; B) Sagittal plane, yellow line illustrating the position of the axial cut in the figure on the side.

**Figure 2:**
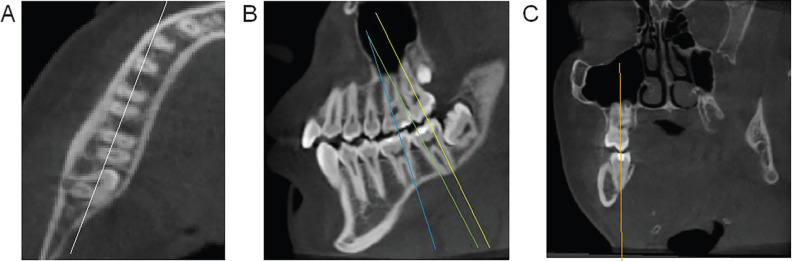
Cone-beam computed tomography images, in three planes, illustrative of the orientation of the sagittal and coronal planes, for subsequent bone measurements. A) Axial section with white line illustrating the positioning of the sagittal plane, it was reoriented, passing through two points in the center of the alveolar process, at the level of the mesial root of the mandibular first molar and the distal root of the mandibular second molar; B) Reoriented sagittal plane, with lines guiding the position of the coronal plane of each of the reference roots; C) Reoriented coronal plane, with a vertical line following the axis of the cervical 2/3 of one of the three roots evaluated per side.

The reorientation of the axial plane followed the alignment of three points: the furcation of the right mandibular first molar, the furcation of the left mandibular first molar and the furcation of the right mandibular second molar (Fig. 1A,B). After this first adjustment, the sagittal plane was reoriented, passing through two points in the center of the alveolar process, at the level of the mesial root of the mandibular first molar and distal root of the mandibular second molar, to identify the mesiodistal direction of the alveolar process in the posterior segment of the mandible (Fig. 2A). The final adjustment was in the coronal plane, following the axis of the cervical 2/3 of each of the three roots evaluated per side (distal root of mandibular first molar and mesial and distal roots of mandibular second molar) (Fig. 2B,C).

**Figure 3:**
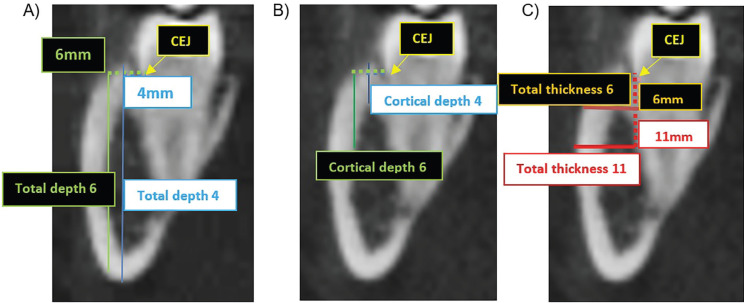
Cone-beam computed tomography images, in the coronal plane, illustrative of the six bone measurements performed in each of the selected roots. A) Coronal plane, with horizontal dotted lines starting from the CEJ (at 4 and 6 mm), guiding the vertical positioning of the total bone depth measurements (cortical bone + medullary bone) in the 2 locations; B) Coronal plane, with horizontal dotted lines starting from the CEJ (at 4 and 6 mm), guiding the vertical positioning of the cortical bone depth measurements at the 2 locations; C) Coronal plane, with vertical dotted lines starting from the CEJ (at 6 and 11 mm), guiding the horizontal positioning of the measurements of the total bone thickness (cortical bone + medullary bone) in the 2 locations.

This procedure generated the coronal sections to assess the total and cortical bone thickness and depth in the buccal region (from the roots mentioned above) of the mandibular molars (Fig. 3A-C). The assessment was performed on both the right and left sides.

The following steps were used for this assessment, following the methodology previously described (9):

- Identification of the buccal cementoenamel junction (CEJ) in each of the coronal sections of the roots;

- Evaluation of the total bone depth (cortical + medullary) in two vertical reference lines located buccally at 4 mm (total depth at 4mm) (Fig. 3A, blue line) and 6 mm (total depth at 6mm) (Fig. 3A, line green) of the CEJ.

- Evaluation of cortical depth, in two vertical reference lines located buccally at 4 mm (cortical depth at 4mm) (Fig. 3B, blue line) and 6 mm (cortical depth at 6mm) (Fig. 3B, green line) from CEJ.

- Evaluation of the total thickness of the buccal bone in two horizontal reference lines located apically 6 mm (total thickness at 6mm) (Fig. 3C, orange line) and 11 mm (total thickness at 11mm) from the (Figure 3C, red line);

All measurements were taken by the same operator.

-Statistical analysis

To assess the intra-rater error, the Wilcoxon test for paired samples and the Intraclass Correlation Coefficient (ICC) were used. The Wilcoxon test allows evaluating the existence of systematic error, checking whether there are significant differences between the initial measurements and the repetitions. The CCI varies between 0 and 1 (the closer to 1, the better the reliability between measurements) and allows evaluating the random error, checking the consistency between the measurements. A non-significant Wilcoxon test (*p*>0.05) and an ICC greater than 0.75 (14) guarantee the reliability of the measurements.

Data normality was assessed using the Shapiro-Wilk test, whose results led to the conclusion that most variables under study do not have a normal distribution. Thus, it was decided to use non-parametric tests.

- Mann-Whitney test to compare the parameters evaluated between independent groups (with the presence of lower third molars vs. with the presence of mandibular third molars; female gender vs. male gender);

- Wilcoxon test for paired samples for comparisons of total depth values with cortical depth values;

- Spearman’s Correlation Coefficient for the analysis of age correlation with the evaluated parameters.

A significance level of 5% was considered, that is, the associations/differences were considered statistically significant when the significance value was less than 0.05 (*p*<0.05). Cases in which the test results were close to statistical significance (*p*<0.10) were also highlighted.

Statistical analysis was performed using the IBM SPSS program, version 24 for Windows (IBM Corp. Released 2016).

## Results

The results of the Wilcoxon tests for comparing the initial measurements and the repetitions were not significant (*p*>0.05) for all variables, indicating that there are no statistically significant differences between the initial measurements and the repetitions performed by the same examiner. The Intraclass Correlation Coefficient (ICC) values were all above 0.90, indicating excellent levels of consistency in the measurements.

Together, the results of the Wilcoxon Test and the CCI guarantee the non-existence of systematic or random error, guaranteeing the consistency and reliability of the measurements.

The results of the study of the correlation with age (Table 2,  Table 2 cont.) showed the existence of negative and statistically significant correlations between age and the variables: Distal Root of 36: total thickness at 6mm (R= -0.263; *p*=0.008), total thickness at 11mm (R= -0.419; *p*<0.001); Mesial root of 37: total thickness at 6mm (R= -0.366; *p*<0.001), total thickness at 11mm (R= -0.204; *p*=0.040); Distal Root of 37: total thickness at 6mm (R= -0.370; *p*<0.001); Distal root of 46: total thickness at 6mm (R= -0.260; *p*=0.008), total thickness at 11mm (R= -0.343; *p*<0.001); Mesial root of 47: total thickness at 6mm (R= -0.388; *p*<0.001), total thickness at 11mm (R= -0.297; *p*=0.002); Distal Root D of 47: total thickness at 6mm (R= -0.446; *p*<0.001), total thickness at 11mm (R=-0.299; *p*=0.002), cortical depth at 6mm (R= -0.210; *p*=0.034). These correlations indicated a tendency for the values of these variables to decrease with increasing age.

**Table 2:**
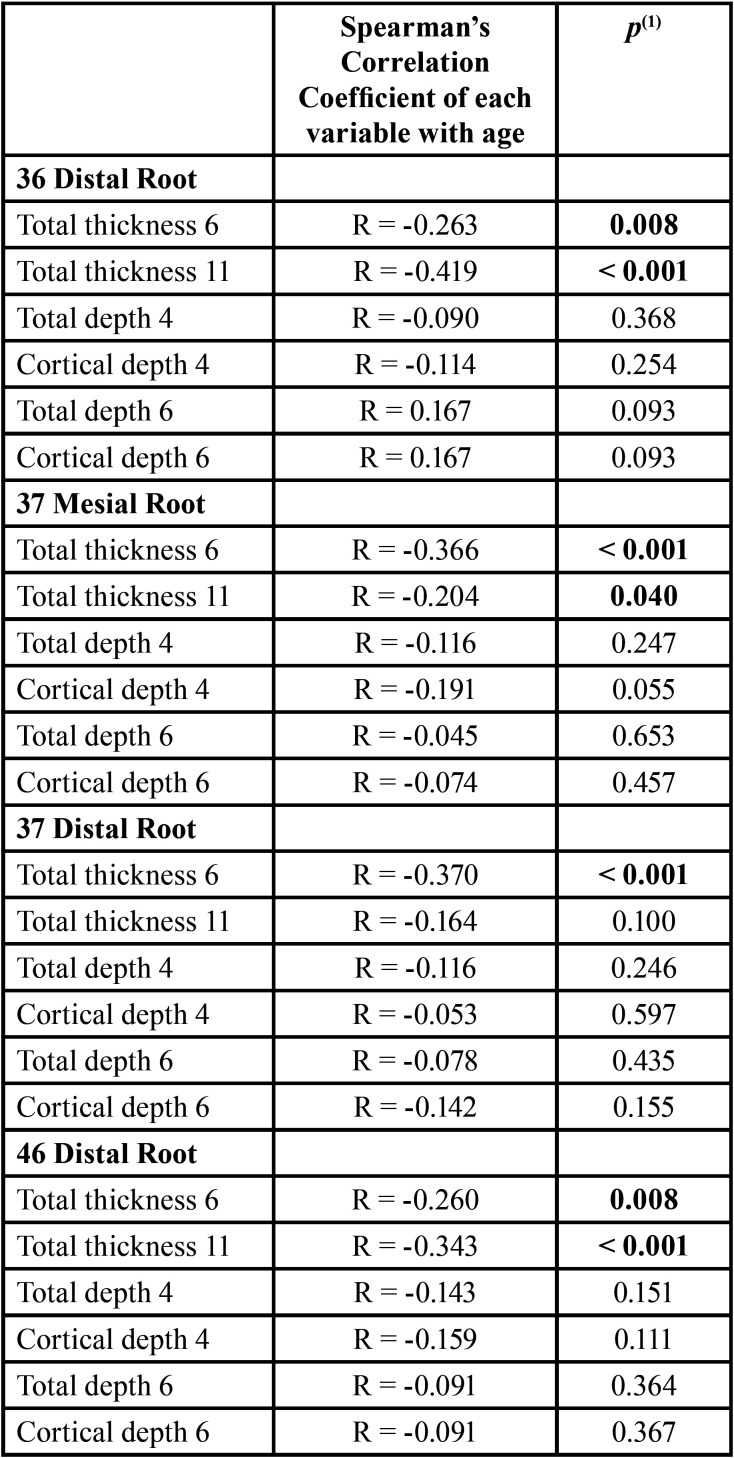
Results of the correlation with age.

**Table 2 cont.**
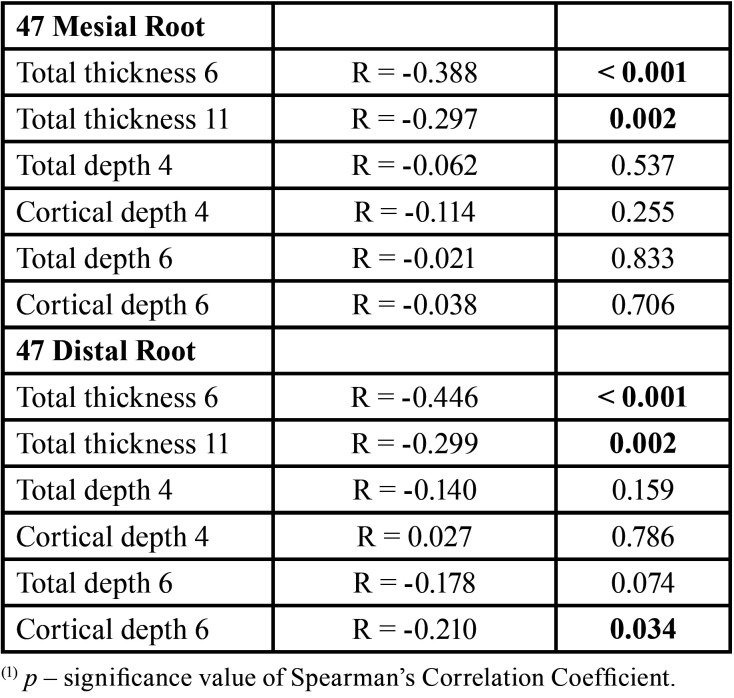
Results of the correlation with age.

The patients with mandibular third molars presented greater mean values in all parameters evaluated, in all roots (Table 3). The differences are statistically significant (*p*<0.05), or close to statistical significance, in the variables: Distal root of 36: total thickness at 11mm (*p*=0.015); Mesial root of 37: total thickness at 6mm (*p*=0.006), total thickness at 11mm (*p*=0.032), total depth at 4mm (*p*=0.042); Distal Root of 37: total thickness at 6mm (*p*=0.003), total thickness at 11mm (*p*=0.016), total depth at 4mm (*p*=0.006), total depth at 6mm (*p*=0.006), cortical depth at 6mm (*p*=0.005); Distal root of 46: total depth at 6mm (*p*=0.086), cortical depth at 6mm (*p*=0.082); Mesial root of 47: total thickness at 6mm (*p*=0.060), total thickness at 11mm (*p*=0.051), total depth at 4mm (*p*=0.078); Distal root of 47: total thickness at 6mm (*p*=0.006), total thickness at 11mm (*p*=0.014), total depth at 4mm (*p*=0.007), total depth at 6mm (*p*=0.056).

**Table 3:**
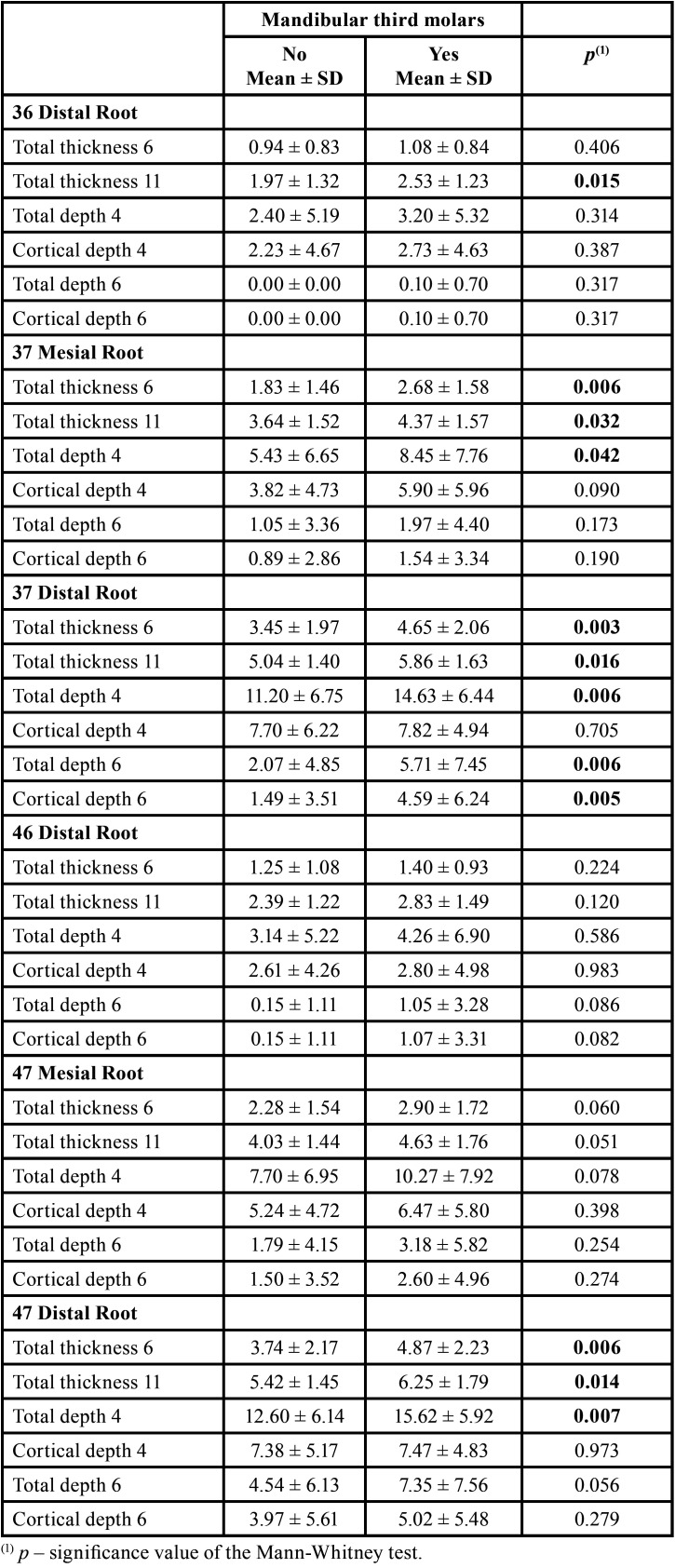
Results of the comparison between patients with and without mandibular third molars.

The results in Table 4 show that the mean values for total depth are higher than the mean values for cortical depth. The differences are not statistically significant, in cases of total depth at 4mm x cortical depth at 4mm (*p*=0.068) and total depth at 6mm x cortical depth at 6mm (*p*=1.000) from the distal root of 36; total depth at 6mm x cortical depth at 6mm (*p*=0.109) from the mesial root of 37; and total depth at 6mm x cortical depth at 6mm (*p*=1.000) from the distal root of 46.

**Table 4:**
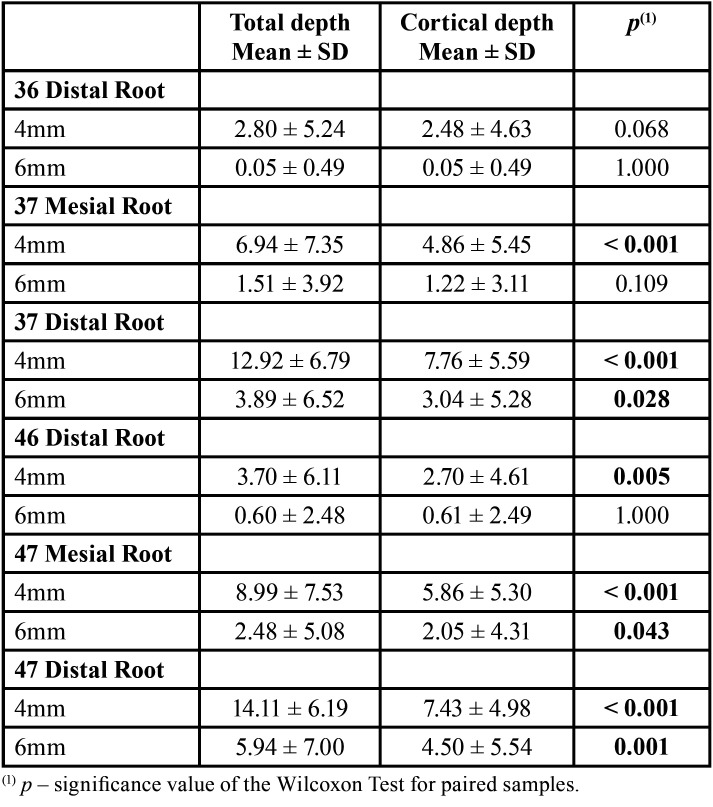
Results of the comparison of the total depth with the cortical depth.

## Discussion

The present study evaluated three relevant bone characteristics in the buccal region of the mandibular molars (total bone thickness, cortical depth and total bone depth) that can help in the planning of extra-alveolar mini-implants. To date, the influence of the presence or absence of mandibular third molars on these bone dimensions has not been investigated. However, there is a limitation in the absence of an evaluation of the previous history, since tomographic images were evaluated with the presence or absence of third molars, which made it impossible to differentiate between absences due to agenesis or extractions and whether there would be any difference in the bone dimensions studied between these two distinct clinical situations.

In the present study, a methodology proposed in the study of Nucera *et al*. (9) was followed, with the difference that measurements were not made in the mesial root region of the mandibular first molars because, due to the little bone prominence in this region, the vast majority of measurements presented values of zero, which would certainly make this region unfeasible as a possible location for the installation of extra-alveolar mini-implants. Other studies (1,6,15) did not present measurements at the level of the mesial root of the mandibular first molar, however, Escobar-Correa *et al*. (5) performed measurements at the mesial root of the first molar, resulting in insufficient mean bone depths and thicknesses for the placement of extra-alveolar mini-implants.

To reach the statistically representative sample size (n=102), respecting the inclusion and exclusion criteria, a large variation was observed in the minimum and maximum ages (13.4 years and 58.7 years respectively), which did not make the results obtained unfeasible, as it represents the growing demand for orthodontic treatment in adolescence and adults over 40 years of age. However, other similar studies used different samples n=32 (7); n=12 (8); n=12 (4); n=30 (1,9), or evaluated specific ethnic groups,(4) which evaluated adults of Asian descent with Class III malocclusion. Other authors used samples with less age variation, such as adults aged 20 to 45 years (16); adults from 21 to 44 years old (7); young people aged 15.3 to 17.7 years (12); children from 8 to 10 years old (8); adults aged 20 to 41 years (9); adolescents from 12.5 to 16.5 years (1) and adults from 19 to 33 years (2), while one study did not mention the age group of the adults in the sample (4). Only the study of Elshebiny *et al*. (1) showed a higher age range than the present study, whose sample of 111 patients ranged in age from 10 to 65 years, however, the authors did not consider any possible influence of this age range on the values obtained for skin thickness cortical bone. Regarding age range, we believe that samples with more homogeneous ages could reduce the effects of remnant growth (for very young patients) and bone resorption (for patients over 50 years old) on the bone measurements studied.

The recently published research of Escobar-Correa *et al*. (5) showed the greatest similarity with the measurements performed in our study, such as bone thickness at 6 mm and 11 mm from the CEJ and total depth at 4 mm and 6 mm from the CEJ. However, they did not measure the depth of the cortical bone, which in our study only the cortical depth at 6mm of the mesial root of 37 showed a statistically significant difference (*p*=0.039). The most recent studies (2,3,5,6,15) on the anatomy of the Mandibular Buccal Shelf Mandibular (MBS) did not show the measurement of the cortical depth, being concerned with the total depth, bone thickness, and in some cases, with the proximity of the mini-implant to the inferior alveolar nerve.

The values of bone thickness, total depth, and cortical depth were related to the patients’ age and showed a tendency to decrease values with increasing age, contradicting the results of Farnsworth *et al*. (17) which observed thicker bone cortical in adult individuals (20 to 45 years, n=26) compared to adolescents (14 to 16 years, n=26). The work of Swasty *et al*. (13) whose sample of 111 individuals had an age range of 10 to 65 years, more similar to our study (13.2 to 59.2 years), did not assess whether the age of the patients would influence the mandibular bone measurements. However, Escobar-Correa *et al*. (5) evaluated the MBS in 64 hemi-arches, observed higher values in all measures studied (bone angulation; bone thickness and depth) for the age group of 16-24 years compared to the other patients, but only the bone angulation and thickness at 6 mm from the CEJ showed a statistically significant difference in relation to age. In the work of Aleluia *et al*. (15), the age of the patients was not mentioned.

The relationship between the presence or absence of mandibular third molars with the variables studied has not been published and showed higher mean values in all parameters evaluated, in all roots, in patients with mandibular third molars when compared to patients with no third molars. However, the differences were statistically significant (*p* < 0.05) for 12 of the 36 measurements performed (Table 5). Among these 12 measurements, the total thickness at 11 mm of the CEJ was statistically significant in four of the six roots evaluated, for patients with third molars: in the distal root of the 36 (2.53 +1.23 mm), in the mesial root of the 37 (4.37 +1.57 mm), at the distal root of 37 (5.86 +1.63 mm) and the distal root of 47 (6.25 +1.79 mm); it was observed that these values are lower than those found by Nucera *et al*. (9) for total thickness at 11mm at the distal root of 36 (4.45 +2.05 mm), at the mesial root of 37 (7.04 +1.65 mm), at the distal root of 37 (7.71 +1.69 mm) and the distal root of 47 (7.88 +1.71 mm) and by Escobar-Correa *et al*. (5), which observed mean values of 3.5 +1.3 mm at the distal root of the first molar, 6.2 +1.7 mm at the mesial root of the second molar and 7.6 +1.6 mm at the distal root of the second molar. Three other measures with a statistically significant difference for patients with third molars were the total thickness at 6 mm from the CEJ (total thickness at 6mm), which in our study presented mean values of 2.68 +1.58 mm at the distal root of the 36; 4.65 +2.06 mm at the distal root of 37; 4.87 +2.23 mm at the distal root of 47. Comparing with the values found by Nucera *et al*. (9) only at the distal root of 36 (1.74 +1.86 mm) that our measure total thickness at 6mm was greater; in the distal root of 37 (5.63 +2.44 mm) and the distal root of 47 (5.57 +2.42 mm) our values were lower. Regarding the study of Escobar-Correa *et al*. (5) for total thickness at 6mm, the mean values were observed of 1.6 +0.8 mm in the distal root of the first molar, 3.3 +1.6 mm in the mesial root of the second molar, and 5.2 +2.1 mm in the distal root of the second molar, and only in the mesial root of the second molar, the value exceeded that found in our study.

Regarding bone depth measurements that showed statistically significant values in patients with third molars, the following means were observed for the total depth at 4 mm from the CEJ: 8.45 +7.76 mm at the mesial root of the 37; 14.63 +6.44 mm at the distal root of the 37; 15.62 +5.92 mm at the distal root of the 47. In other work of Nucera *et al*. (9) the values for these three measurements were higher, 18.62 +4.89 mm; 19.98 +3.22mm and 19.84 +3.28mm, respectively. The values found by Escobar-Correa *et al*. (5) were also higher, 15.5 +5.3 mm in the mesial root of the second molar and 18.7 +3.8 mm in the distal root of the second molar. The last two measurements that showed a statistically significant difference between patients with and without third molars were the total depth at 6mm from the CEJ and the cortical depth at 6 mm from the CEJ, both in the distal root of the 37. The latter was the only measure of cortical depth that showed a statistically significant difference between patients with and without third molars. In the present study, a mean of 5.71 +7.45 mm was observed for total depth at 6mm and 4.59 +6.24 mm for cortical depth at 6mm in the distal root of 37, below the values found by Nucera *et al*. (9) of 17.44 +6.51 mm and 6.99 +6.31 mm for the respective measurements, especially for total depth at 6mm. As Escobar-Correa *et al*. (5) did not measure the depth of the cortical bone, they observed an average of 13.9+-6.2 mm for prof tot 6, above the values found in this study and below those mentioned by Nucera *et al*. (9)

In the present study, the correlation between cortical bone depth and total depth was performed, which is only possible in studies that evaluated the anatomy of the MBS aimed at the use of extra-alveolar mini-implants. Even with a similar methodology, Nucera *et al*. (9) did not verify this relationship. In more recent studies (Liu *et al*. (2); Gandhi *et al*. (6); Vargas *et al*. (3); Costa *et al*. (15); Escobar-Correa *et al*. (5)), the measure of the cortical depth was not performed, making this comparison impossible. As the total measures complement the cortical ones, higher and statistically significant values were observed for the total measures, both at 4 mm and 6 mm from the CEJ, except for total depth at 4mm vs. cortical depth at 4mm (*p*=0.068) and total depth at 6mm vs. cortical depth at 6mm (*p*=1.000) from the distal root of 36; total depth at 6mm vs. cortical depth at 6mm (*p*=0.109) from the mesial root of 37; and total depth at 6mm vs. cortical depth at 6mm (*p*=1.000) from the distal root of the 46. These statistically non-significant correlation results could be related to the fact of lower bone availability in the region of first molars and when it was measured at 6 mm buccally to the CEJ, resulting in measurements of value zero for both total depth and cortical depth measurements (which occurred mainly in the region of first molars), or resulting in locations where the measurement was taken very close to the buccal edge, where there is only cortical bone, making total depth measurements and cortical depth identical (most common in measurements at 6 mm buccally to the CEJ).

In several measurements, especially those located in the second molars, high values of the standard deviation of the means were observed. This is because some patients show values with wide variation from the mean, above or below, indicating that although bone measurements generally increase in the posterior direction, there are significant anatomical variations between patients and it is necessary to be careful when planning the installation of mini-implants in the region of the mandibular buccal shelf (3).

The most favorable location for the placement of mini-implants in the region of the mandibular buccal shelf was the region of the distal root of the mandibular second molars, as it approached the minimum value of 5 mm (9) of horizontal bone thickness at 6 mm from the CEJ and values above the minimum value at 11 mm from the CEJ (1.7 mm safety distance to the root; 1.6 mm mini-implant diameter and 1.7 mm safety distance from the buccal bone cortex) and values greater than the minimum of 6 mm vertical bone depth (which represents the minimum mini-implant length). Although the values observed in the present study are lower than those reported by other studies (1,4,9), our study agrees with the most favorable location for the installation of mini-implants in the region of the mandibular buccal shelf (1,9). However, for other authors, the safest place to install mini-implants in the MBS was the region between the first and second molars (2,4).

A systematic review with meta-analysis (18) found a positive association between the primary stability of mini-implants and the thickness of the alveolar cortical bone, and several studies have tried to evaluate the thickness of the cortical bone through CBCTs in different favorable locations for installing these devices. Most of these studies looked at sites for placing the mini-implants between roots. Regarding studies evaluating the thickness of the alveolar cortical bone, higher cortical values were observed in adults when compared to adolescents and there was a great variation between different regions in the maxilla and mandible (17), an increase in the thickness of the mandibular cortical bone was observed. As the distance from the alveolar crest increases (7), and a smaller thickness of cortical bone in the central region between two teeth in relation to the portion more adjacent to the roots was described (10).

In line with the present research, other similar studies have shown thickness of buccal bone cortical above 2 mm in the region of the second molars (1,9), which would indicate the previous perforation of this cortical, to reduce the insertion torque of these screws, decreasing the risk of fractures.

The results of the present study agree with the literature that in the region of the distal root of mandibular second molars, both bone thickness and total and cortical depth are satisfactory for the placement of extra-alveolar mini-implants in the mandibular buccal shelf; however, relatively high values were observed in the standard deviation, which denotes an important difference, especially for values below the mean, which would make this location unfeasible as an installation site. Three-dimensional exams would be important for planning mini-implants in the MBS only in the region of mandibular first molars, as they presented lower values for bone and cortical thickness in relation to the region of mandibular second molars, with higher values of standard deviation. According to the same authors, the region of the mandibular second molars presented a consistent pattern of bone dimensions sufficient for the placement of mini-implants, where the routine use of three-dimensional exams would not be indicated (1).

The following clinical considerations are important: when the adolescent/adult patient undergoes orthodontic treatment, it is often necessary to use bone anchorage in the posterior region to optimize the correction of the anterior and posterior relationship, however, it is not always possible to install mini-implants between the roots of the mandibular molars, as well as in the buccal region of these teeth. As the tooth is dependent on the alveolar bone, the present study evaluated the influence of the third molar on bone thickness and height of the buccal region of the second and first molars. There is a tendency for a decrease in buccal bone thickness with increasing age, and both bone thickness and total bone depth in the buccal region of the mandibular molars were greater in patients with mandibular third molars because the buccal bone thickness of the mandibular molars increased in the posterior and apical direction. Thus, dentists can instruct patients not to perform prophylactic removal (absence of disease) routinely, even when another professional colleague indicates, because the more bone in this region, the safer will be the placement of mini-implants in the buccal region of the mandibular molars.

## Conclusions

The mean values for total bone thickness, total depth and cortical bone in the buccal region of the mandibular molars were greater for patients with mandibular third molars. The buccal bone thickness of the mandibular molars increased in the posterior and apical directions. Both the cortical depth and the total depth (cortical bone + medullary bone) in the buccal region of mandibular molars showed a progressive increase in the posterior direction, with higher values at 4 mm from the CEJ compared to measurements taken at 6 mm.
